# Supplementary material for: IL-10 and IL-17 as Progression Markers of Syphilis in People Living with HIV: A Systematic Review
Source: Biomolecules. 2022 Oct 13;12(10):1472. doi: 10.3390/biom12101472 (PMC9599307; doi:10.3390/biom12101472)

Supplementary Table S1. Systematic review of the immune response against Treponema pallidum subs pallidum.

			Syphilis		
**Stage**	**Cells involved**	**Response**	**Cytokines/ Antibodies**	**Transcription Factors**	**References**
Primary	Th1	High	IFN-γ / TNF	STAT1 / T-bet	[10,13,15]
	Th2	Low	IL-4	GATA3	[13]
	Trex	High	IL-10 / TGF-β	Foxp3	[9,13,18]
	Th17	Low	IL-17Ra	ROR-γt	[9,24]
	Mo	High	--	--	[5,10]
	DC	High	--	--	[5,30,36]
Secondary	NK	High (Skin)	IFN-γ	RORa/GATA3	[5]
	Th1	High	IFN-γ / TNF	STAT1/T-bet	[10,13,15]
	Trex	High	IL-10 / TGF-β	Foxp3	[13,9]
	CD8	Low	IFN-γ Granzime B	T-bet	[5,13]
	DC	Low	--	--	[5,30]
Tertiary	NK	High (blood)	IFN-γ	RORa/GATA3	[5]
	Th17	High	IL-17Ra	ROR-γt	[18,24,30]
	B	High	IgM / IgG	NFkb	[34,35]
	CD8	High	IFN-γ	T-bet	[13]

Supplementary Table S2. Systematic review of the immune response in people living with HIV co-infected with Treponema pallidum subs pallidum.

**Syphilis in persons living with HIV**				
Stage	**Cells**	**Response**	**Results**	**References**
**Primariy**	CD4	Low	Decrease of 85% and 80% in people with ART and without ART respectively.<br><br>Primary co-infection was associated with an increase in IL-10. Both IL-10 and TNF decreased after syphilis treatment.	[22,23]
	Tγδ	High	Greater number of IL-17 producing Tγδ cells and up to 8 times greater IFN-γ production.	[24]
	T rex	High	Greater number of Foxp3+Helios+CD25+ T rex compared with healthy donnors.	[25]
	Intermediate Monocytes	High	Higher antigen presentation HLA-DR.<br>Higher migration with CCR2 and CX3CR1 chemokines.	[26]
**Secondary**	CD4	No difference *versus* healthy donors	Nine times higher concentration of IL-10 was reported.	[27]
**Tertiary**	CD4	Low	"Lymphopenia" as a predictor of neurosyphilis.	[28]

Supplementary Figure S1. Kinetics of the progression of Syphilis vs. HIV.

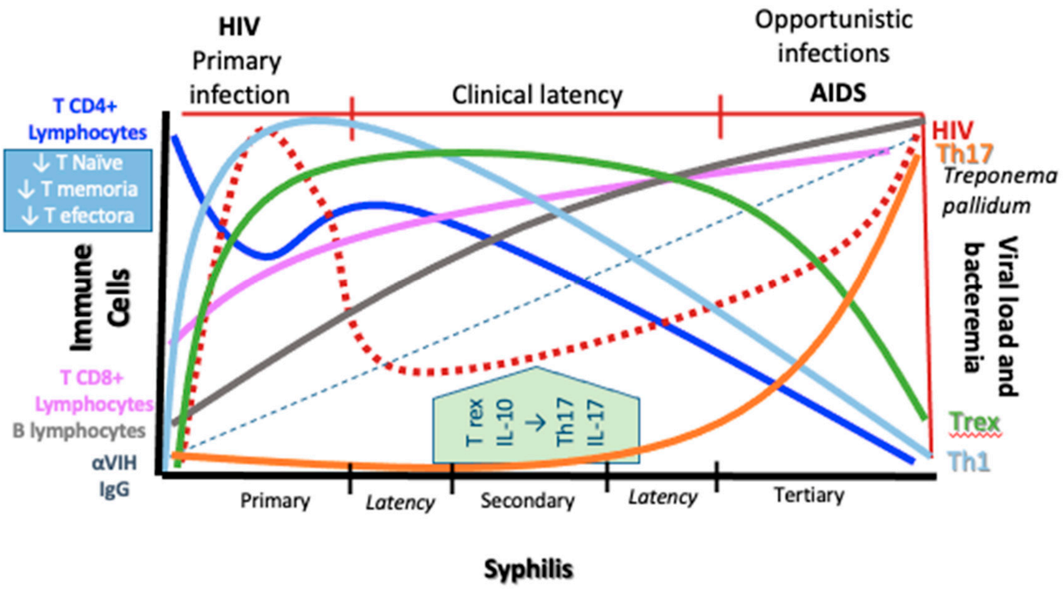

Supplement: Supplementary file 1 [file biomolecules-12-01472-s001.zip › biomolecules-1943973-supplementary.pdf]
